# Author Correction: Absolute measurement of cellular activities using photochromic single-fluorophore biosensors and intermittent quantification

**DOI:** 10.1038/s41467-023-37856-4

**Published:** 2023-04-17

**Authors:** Franziska Bierbuesse, Anaïs C. Bourges, Vincent Gielen, Viola Mönkemöller, Wim Vandenberg, Yi Shen, Johan Hofkens, Pieter Vanden Berghe, Robert E. Campbell, Benjamien Moeyaert, Peter Dedecker

**Affiliations:** 1 Department of Chemistry, KU Leuven, Leuven, Belgium; 2 Department of Chemistry, University of Alberta, Edmonton, Canada; 3 Department of Chronic Diseases Metabolism and Ageing, KU Leuven, Leuven, Belgium; 4 Department of Chemistry, University of Tokyo, Tokyo, Japan

**Keywords:** Fluorescence imaging, Ca2+ imaging, Fluorescent proteins, Imaging and sensing, Wide-field fluorescence microscopy

Correction to: *Nature Communications* 10.1038/s41467-022-29508-w, published online 06 April 2022

In this article the grant number 101033291, COSMO relating to European Commission fellowship for Anaïs C. Bourges was omitted. The original article has been corrected.

