# Supplementary material for: Absolute measurement of cellular activities using photochromic single-fluorophore biosensors and intermittent quantification
Source: Nat Commun. 2022 Apr 6;13:1850. doi: 10.1038/s41467-022-29508-w (PMC8986857; doi:10.1038/s41467-022-29508-w)
Supplement: Supplementary file 2 — Reporting Summary [file 41467_2022_29508_MOESM2_ESM.pdf]

nature portfolio

Corresponding author(s): Peter Dedecker

Last updated by author(s): Mar 14, 2022

Reporting Summary

Nature Portfolio wishes to improve the reproducibility of the work that we publish. This form provides structure for consistency and transparency in reporting. For further information on Nature Portfolio policies, see our Editorial Policies and the Editorial Policy Checklist.

Statistics

For all statistical analyses, confirm that the following items are present in the figure legend, table legend, main text, or Methods section.

n/a	Confirmed	
☐	☒	The exact sample size (*n*) for each experimental group/condition, given as a discrete number and unit of measurement
☐	☒	A statement on whether measurements were taken from distinct samples or whether the same sample was measured repeatedly
☒	☐	The statistical test(s) used AND whether they are one- or two-sided<br>*Only common tests should be described solely by name; describe more complex techniques in the Methods section.*
☒	☐	A description of all covariates tested
☒	☐	A description of any assumptions or corrections, such as tests of normality and adjustment for multiple comparisons
☐	☒	A full description of the statistical parameters including central tendency (e.g. means) or other basic estimates (e.g. regression coefficient) AND variation (e.g. standard deviation) or associated estimates of uncertainty (e.g. confidence intervals)
☒	☐	For null hypothesis testing, the test statistic (e.g. *F*, *t*, *r*) with confidence intervals, effect sizes, degrees of freedom and *P* value noted<br>*Give P values as exact values whenever suitable.*
☒	☐	For Bayesian analysis, information on the choice of priors and Markov chain Monte Carlo settings
☒	☐	For hierarchical and complex designs, identification of the appropriate level for tests and full reporting of outcomes
☒	☐	Estimates of effect sizes (e.g. Cohen's *d*, Pearson's *r*), indicating how they were calculated

Our web collection on statistics for biologists contains articles on many of the points above.

Software and code

Policy information about availability of computer code

Data collection	Measurements with the Tecan Saffire II were controlled through the Microsoft Excel based XFLUOR4SAFIREII (V4.62b). All other measurements were conducted on setups with homemade software for controlling these setups.
Data analysis	All software used for data analysis was written and compiled in Igor Pro, a software developed and distributed by WaveMetrics (https://www.wavemetrics.com). The code was tested on Igor Pro version 8.04, run on Microsoft Windows 10 Education.<br>A detailed description of the analysis methodologies can be found in the manuscript. Minimal code examples for PEAQ and iPEAQ are provided on Zenodo at https://doi.org/10.5281/zenodo.5939766.

For manuscripts utilizing custom algorithms or software that are central to the research but not yet described in published literature, software must be made available to editors and reviewers. We strongly encourage code deposition in a community repository (e.g. GitHub). See the Nature Portfolio guidelines for submitting code & software for further information.

Data

Policy information about availability of data

All manuscripts must include a data availability statement. This statement should provide the following information, where applicable:

- Accession codes, unique identifiers, or web links for publicly available datasets
- A description of any restrictions on data availability
- For clinical datasets or third party data, please ensure that the statement adheres to our policy

The datasets generated during and/or analysed during the current study are available from Zenodo at https://doi.org/10.5281/zenodo.5939766. Source data are provided with this paper.

Field-specific reporting

Please select the one below that is the best fit for your research. If you are not sure, read the appropriate sections before making your selection.

☒ Life sciences ☐ Behavioural & social sciences ☐ Ecological, evolutionary & environmental sciences

For a reference copy of the document with all sections, see nature.com/documents/nr-reporting-summary-flat.pdf

Life sciences study design

All studies must disclose on these points even when the disclosure is negative.

Sample size	For each independent histamine stimulation experiment, a 35mm glass-bottom dish sample was imaged at one sample position. Multiple regions of interest (ROI) corresponding to single cells were determined. Each experiment was repeated with multiple dishes over multiple days.<br>For the cell titrations, a 35mm glass-bottom dish sample was imaged at two sample position. Multiple regions of interest (ROI) corresponding to single cells were determined. This was repeated with multiple dishes.<br>No sample size calculation was performed. Sample sizes where considered sufficient, if consistent measurement results were being found for a minimum amount of three cell dishes.<br>Further information can be found in the Methods section of the paper.
Data exclusions	Only cells in the field of view with sufficient brightness and a pronounced photochromism were considered for the analysis. Further, the adhesion of the cells to the dish for the whole duration of the experiment was verified during the cell selection for the data analysis. Other selection criteria, i.e. for co-culture experiments are described in the Methods section.
Replication	The Methods section gives inside into the replication of the different conducted experiments.<br>The histamine stimulation experiments were successful with the exception of transfection issues.<br>The cell titration experiments generally showed a similar trend, if no transfection issues occured.
Randomization	N/A (Only one group was considered for the performed experiments containing cells transfected with the studied biosensor GCaMP6s-Q or both GCaMP6s-Q and Cameleon YC2.60 simultaneously.)
Blinding	N/A (The samples were treated equally. Exclusion criteria were mentioned above.)

Reporting for specific materials, systems and methods

We require information from authors about some types of materials, experimental systems and methods used in many studies. Here, indicate whether each material, system or method listed is relevant to your study. If you are not sure if a list item applies to your research, read the appropriate section before selecting a response.

Materials & experimental systems

n/a	Involved in the study	
☒	☐	Antibodies
☐	☒	Eukaryotic cell lines
☒	☐	Palaeontology and archaeology
☒	☐	Animals and other organisms
☒	☐	Human research participants
☒	☐	Clinical data
☒	☐	Dual use research of concern

Methods

n/a	Involved in the study	
☒	☐	ChIP-seq
☒	☐	Flow cytometry
☒	☐	MRI-based neuroimaging

Eukaryotic cell lines

Policy information about cell lines

Cell line source(s)	HeLa (ATCC-CCL-2) were acquired at ATCC and were regularly replaced from frozen stocks.
Authentication	None of the cell lines used were authenticated.
Mycoplasma contamination	The cell lines were not tested for mycoplasma contamination.
Commonly misidentified lines (See ICLAC register)	No commonly misidentified lines were used.
